# Real-time MR tracking of AAV gene therapy with βgal-responsive MR probe in a murine model of GM1-gangliosidosis

**DOI:** 10.1016/j.omtm.2021.08.003

**Published:** 2021-08-26

**Authors:** Toloo Taghian, Ana Rita Batista, Sarah Kamper, Michael Caldwell, Laura Lilley, Hao Li, Paola Rodriguez, Katerina Mesa, Shaokuan Zheng, Robert M. King, Matthew J. Gounis, Sophia Todeasa, Anne Maguire, Douglas R. Martin, Miguel Sena-Esteves, Thomas J. Meade, Heather L. Gray-Edwards

**Affiliations:** 1 Horae Gene Therapy Center, University of Massachusetts Medical School, Worcester, MA 01605, USA; 2 Department of Neurology, University of Massachusetts Medical School, Worcester, MA 01605, USA; 3 Departments of Chemistry, Molecular Biosciences, Neurobiology and Radiology, Northwestern University, Evanston, IL 60208, USA; 4 Department of Radiology, University of Massachusetts Medical School, Worcester, MA 01655, USA; 5 Scott-Ritchey Research Center, Auburn University, Auburn, AL 36849, USA; 6 Department of Biomedical Engineering, Worcester Polytechnic Institute, Worcester, MA 01609, USA

**Keywords:** AAV gene therapy, magnetic resonance imaging, enzyme-activated contrast agent probes, GM1 gangliosidosis, MR tracking of enzyme expression

## Abstract

Transformative results of adeno-associated virus (AAV) gene therapy in patients with spinal muscular atrophy and Leber’s congenital amaurosis led to approval of the first two AAV products in the United States to treat these diseases. These extraordinary results led to a dramatic increase in the number and type of AAV gene-therapy programs. However, the field lacks non-invasive means to assess levels and duration of therapeutic protein function in patients. Here, we describe a new magnetic resonance imaging (MRI) technology for real-time reporting of gene-therapy products in the living animal in the form of an MRI probe that is activated in the presence of therapeutic protein expression. For the first time, we show reliable tracking of enzyme expression after a now in-human clinical trial AAV gene therapy (ClinicalTrials.gov: NTC03952637) encoding lysosomal acid beta-galactosidase (βgal) using a self-immolative βgal-responsive MRI probe. MRI enhancement in AAV-treated enzyme-deficient mice (GLB-1^−/−^) correlates with βgal activity in central nervous system and peripheral organs after intracranial or intravenous AAV gene therapy, respectively. With >1,800 gene therapies in phase I/II clinical trials (ClinicalTrials.gov), development of a non-invasive method to track gene expression over time in patients is crucial to the future of the gene-therapy field.

## Graphical abstract

**Figure:**
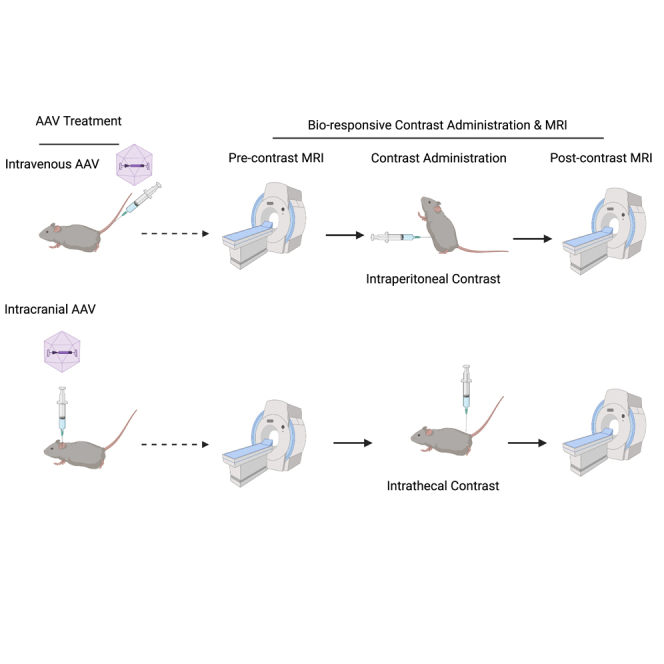

## Teaser

We describe a bio-responsive contrast agent that informs on the biodistribution and durability of gene expression in the lysosomal storage disease, GM1 gangliosidosis. We illustrate the sensitivity of this contrast agent to detect β-galactosidase enzyme in the central nervous system and periphery of an AAV-GLB-1-treated mouse model of GM1.

## Introduction

We and others^1^, ^2^, ^3^, ^4^, ^5^, ^6^, ^7^, ^8^, ^9^, ^10^, ^11^, ^12^, ^13^, ^14^, ^15^, ^16^, ^17^, ^18^, ^19^, ^20^ have shown dramatic efficacy of adeno-associated virus (AAV) gene therapy in animal models including encouraging results in patients. The overwhelming success of these studies has resulted in an explosion in the number of academic- and biotech-led AAV programs in the last few years, with >1,800 ongoing gene-therapy trials^21^, ^22^, ^23^, ^24^, ^25^, ^26^, ^27^, ^28^, ^29^, ^30^ (ClinicalTrials.gov). For a number of these AAV gene-therapy clinical trials, initial dosing has yielded modest results, with increased dosing required for maximal benefit. This situation is a significant hindrance, leaving physicians guessing which organs or tissues are effectively treated. Without an effective real-time diagnostic, the disconnect between dosage control and therapeutic response threatens the clinical progress and approval of urgently needed gene-based therapeutics. Additionally, the need for non-invasive, disease-specific biomarkers is not limited to gene therapy but applies to any therapy that augments the targeted enzyme activity, including enzyme replacement therapy (ERT), transcriptional read-through agents, and/or chaperone therapies.^31^ Effectiveness of gene therapies often correlates well with specific enzymatic activities such as lysosomal β-galactosidase (βgal) activity in GM1 gangliosidosis (GM1). Therefore, the real-time tracking of βgal activity is tantamount to evaluation of a therapeutic response in GM1 and in the case of magnetic resonance (MR) contrast agents,^32^^,^^33^ can be spatially resolved to any organ in the body, including the brain.

## Results

In this report, we show detection of lysosomal acid βgal activity using MR imaging (MRI) in βgal-deficient GM1 mice after AAV gene transfer and correlation of enhancement with enzymatic activity *ex vivo*. This gene-therapy approach has shown remarkable efficacy in GM1 mice,^34^^,^^35^ and GM1 cats^6^ and is now in human clinical trials (ClinicalTrials.gov: NCT03952637).^22^ This new contrast agent holds promise to inform on AAV gene-therapy efficacy in GM1 patients, allowing for non-invasive, long-term assessment of durability of gene expression. Additionally, results from this study apply to a large portion of the gene-therapy field, since this class of agents can be rapidly adapted to nearly any disease with an enzymatic deficiency.

**Figure 1:**
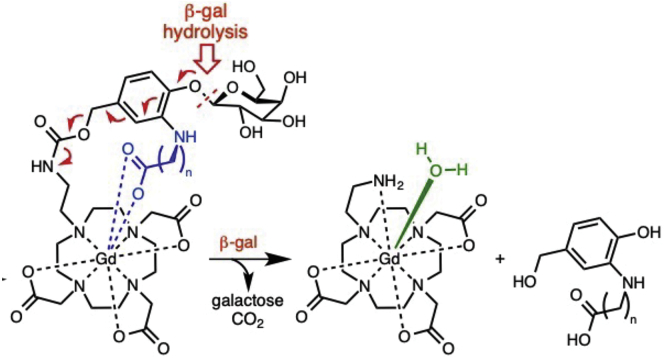
βgal-responsive contrast agent Self-immolative magnetic resonance (MR) agents incorporating a coordinating carboxylate (blue, where n = 5). Gd(III) coordination by this functionality effectively prohibits water access to Gd(III) creating an inactive, or dark, agent by MR imaging. Hydrolysis of the glycoside by βgal results in an electron cascade (red) that provides an open coordination site for water to bind to Gd(III). There is a 90% increase in the observed relativity post-enzyme cleavage.^32^

**Figure 2:**
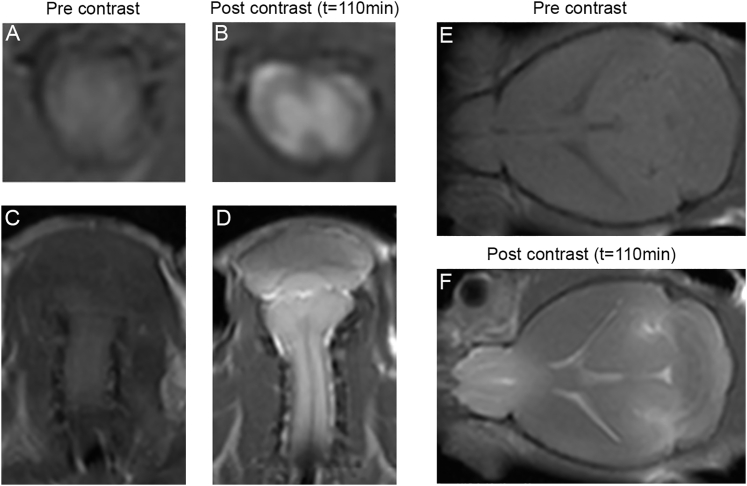
Activation of βgal-responsive contrast agent in the central nervous system Representative MR images showing enhancement of the activated βgal-responsive MR contrast agent in the spinal cord (A and B) and hindbrain cervical cord (C and D). There is strong enhancement of gray matter and surrounding CSF after intrathecal administration. Coronal image of mouse brain illustrating global enhancement throughout the brain parenchyma and CSF at t = 110 min (E and F).

**Figure 3:**
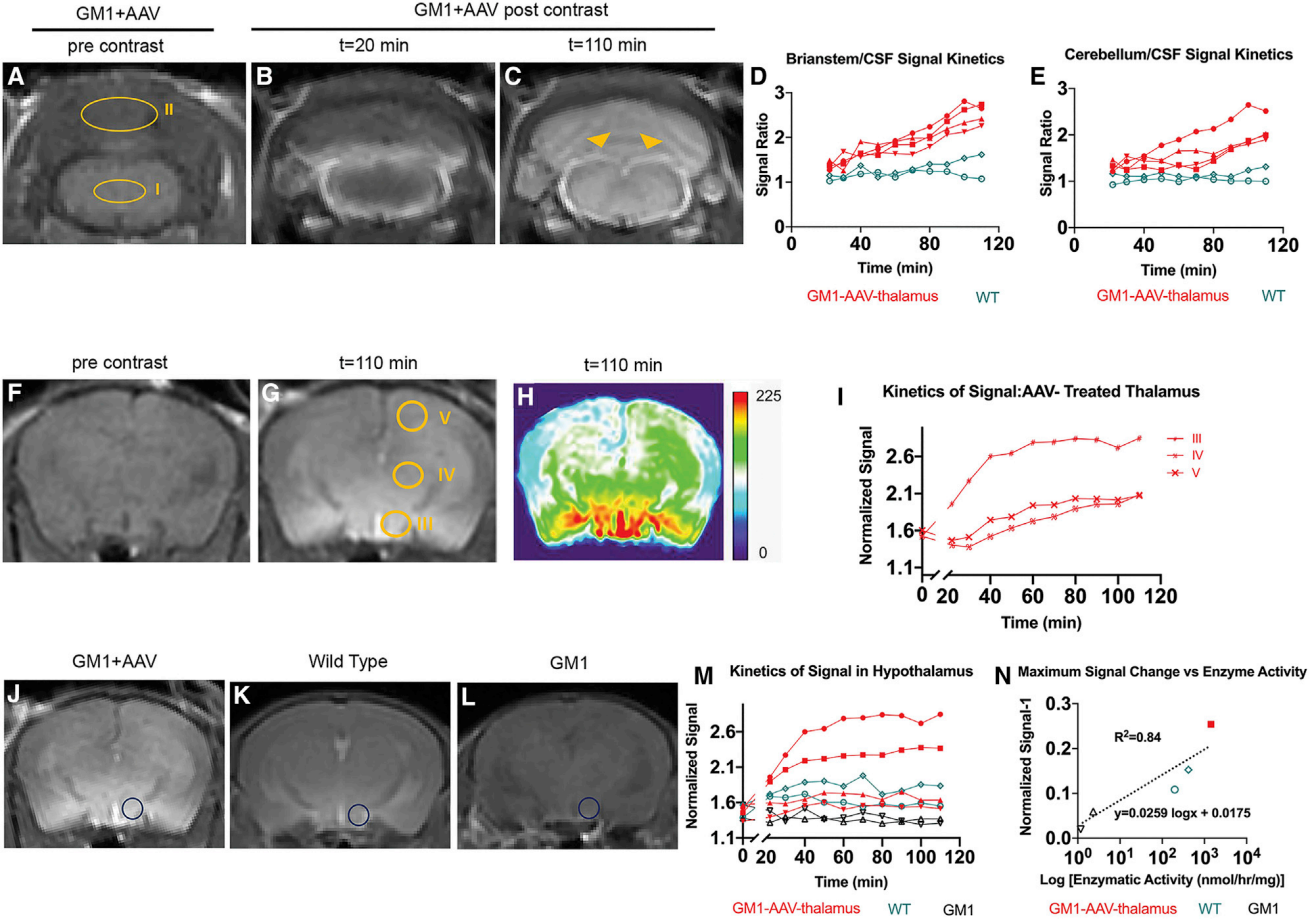
Kinetics of βgal-responsive contrast agent activation in the brain Activation of βgal-responsive MR contrast agent in the brain after intracranial AAV gene therapy. (A−E) Penetration of the probe from CSF to parenchyma over time in brainstem (I) and cerebellum (II). Arrows indicate enhanced CSF surrounding the brainstem. (F−I) Signal changes were quantified in areas over time (III−V: hypothalamus, thalamus, cortex, respectively). Pseudo-color image shows signal enhancement (H). (J−L) Signal enhancement in ventral region of thalamus, 110 min after contrast injection in (J) AAV-treated GM1 mice (filled red squares, circles, and triangles; n = 4), (K) WT (wild type; open green diamonds and circles; n = 2), and (L) GM1 mice (open black triangles; n = 2). (M) Plot of change in normalized signal over time in individual mice. While AAV-treated GM1 and WT mice show signal increase, no signal increase was observed in untreated GM1 mice. (N) MR signal enhancement shows strong correlation with enzyme activity. Color-coded geometrical shapes in (N) correspond to individual mice shown in (M). Three AAV-treated GM1 mice (closed red circle and triangles in M are not represented in N because they didn’t recover from anesthesia). Signal intensity is normalized to muscle signal (Figure S2). Confirmation of successful contrast administration is shown in Figure S3.

To determine the capability of this probe to inform on enzyme distribution after AAV gene therapy, GM1 mice were injected with an AAV9-βgal intracranially (10^10^ vg, in the thalamus, unilateral n = 4, or intravenously [i.v.] 3 × 10^11^ vg, n = 4), as we previously described.^34^^,^^35^ After intrathecal injection of the βgal-responsive contrast agent (Figure 1), intracranially AAV-treated GM1 mice exhibit signal enhancement in cerebral spinal fluid (CSF) and parenchyma (Figure 2; Video S1). Enhancement of CSF occurred rapidly after intrathecal administration (<20 min) with an increasing ratio of parenchymal signal/CSF signal that indicates penetration of the contrast agent from the CSF into the brain tissue (Figures 3A−3E). Distribution of the probe within the brain was greatest on the ventral aspect with a gradient projecting dorsally (Figures 3F−3M; Video S2). The dynamic range is linear and extends to at least 3 logs with a strong correlation between maximum change in enhancement and βgal enzyme activity within the brain (R^2^ = 0.84; Figure 3N). (Three AAV-treated and one wild-type mouse died overnight after MRI and are not included in correlation.) Intracranially AAV-treated mice that received intrathecal administration of the contrast agent showed between a 9.5% and 45.2% increase in MR signal. The MR enhancement greatly correlates with enzyme activity in other brain regions as shown in Figure S4 including frontal (R^2^ = 0.94), striatum (R^2^ = 0.93), and brainstem (R^2^ = = 0.79) but not in midbrain (R^2^ = 0.5) and cerebellum (R^2^ = 0.35).

Video S1. Activation of βgal-responsive contrast agent in the spinal canal and hindbrain

Video S2. Activation of βgal-responsive contrast agent in the brain parenchyma

**Figure 4:**
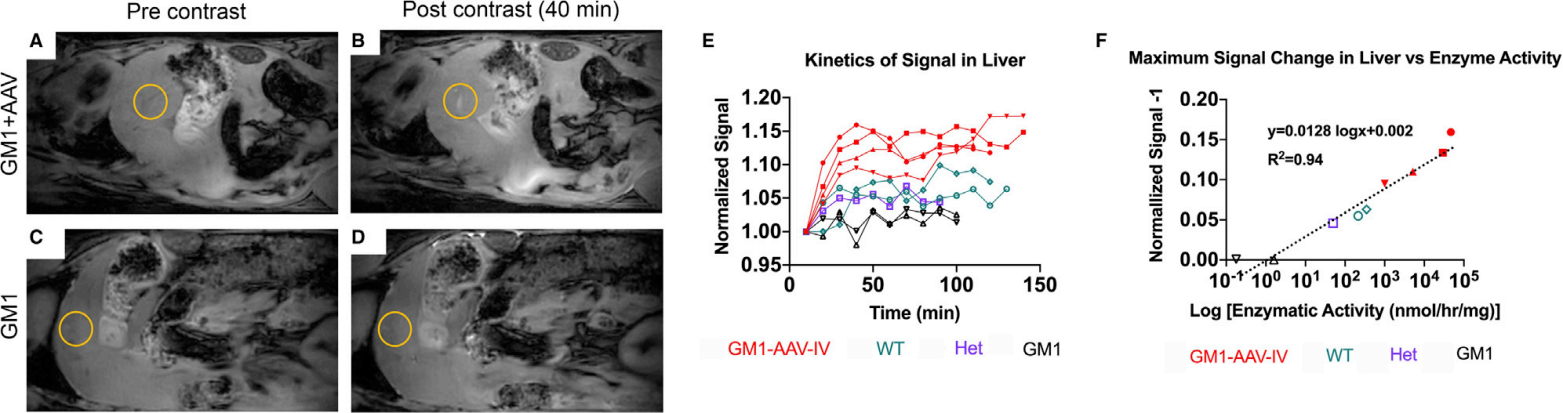
Kinetics of βgal-responsive contrast agent activation in the liver Activation of βgal contrast agent in liver of AAV-treated GM1 mice (A and B) versus GM1 mice (C and D). (E) Normalized signal of liver was quantified in AAV-treated GM1 mice (filled red squares, circles, and triangles; n = 4), WT (open green diamonds and circles; n = 2), Het (heterozygous; open purple square; n = 1), and GM1 mice (open black triangles; n = 2) over time. Criteria for liver ROI selection is described in Figure S5. (F) Correlation of MR signal enhancement with βgal enzyme assay activity (R^2^ = 0.94). Color-coded geometrical shapes in (F) correspond to individual mice shown in (E). Signal was normalized to liver signal right after probe injection.

The βgal-responsive contrast agent was then evaluated in GM1 mice treated systemically with AAV9-βgal. Signal intensity in the liver after contrast agent administration (intraperitoneal [i.p.]) rose sharply over the first 40−50 min, after which it plateaued for the duration of the MRI (>2 h; Figures 4A−4E; Video S3) and showed between a 10% and 15% increase in MR signal. Enzymatic activity correlated with enhancement and extended to at least 4 logs (Figure 4F; R^2^ = 0.94). In the kidney, enhancement followed a similar pattern, except we noted enhancement in untreated GM1 mice, likely due to other enzymes with βgal activity, expressed in renal tissue, as previously reported in GM1 mice.^36^ In the kidney, there was no apparent correlation between βgal activity and probe enhancement (Figure S1), suggesting that renal clearance of the agent prevents assessment of renal enzyme activity. The probe enhancement in the liver of AAV-treated GM1 mice is consistent with that previously shown in the abdomen of mice overexpressing LacZ (bacterial cytoplasmic βgal).^32^

Video S3. Activation of βgal-responsive contrast agent in the liver

The strongest signal enhancement was observed in organs, or sub-structures, where the greatest enzymatic activity has been documented for a particular AAV delivery route.^34^^,^^35^ For example, after i.v. administration of AAV9-βgal, the liver is the organ with the greatest transduction and subsequent enzymatic activity. Enhancement of the liver was pronounced in AAV-treated animals and correlated with enzymatic activity (R^2^ = 0.94). Peritoneal fluid enhancement was noted in all i.v. AAV-treated animals, which is not surprising after i.p. administration, since βgal is a lysosomal enzyme that is secreted when overexpressed after gene transfer. Whereas i.p. injection is an accepted surrogate for intravascular administration in rodents, we anticipate that i.v. injection will increase the kinetics and distribution of this agent. Due to the nature of the contrast agent, it is unlikely that it will cross the blood brain barrier to detect βgal activity in the brain after vascular administration.

To evaluate the sensitivity of the contrast agent in detecting enzyme at wild-type concentrations, we included wild-type and heterozygous mice in the study and quantified the MR signal enhancement as shown in Figures 3 and 4. Wild-type mice receiving intrathecal administration of the contrast agent showed up to an 8% increase in the MR signal, quantified in the hypothalamus as shown in Figures 3K and 3M. Wild-type and heterozygous mice that received the contrast intraperitonially showed a 5%–9% increase in signal enhancement in the liver as shown in Figure 4E. In knockout mice, the signal fluctuates and does not show a steady increase over time (Figures 3L and 3M and Figures 4D and 4E).

## Discussion

After intrathecal administration, compounds initially pool under the ventral aspect of the brain and penetrate parenchyma via the Virchow-Robin spaces along cerebral blood vessels (e.g., anterior, middle, and posterior cerebral arteries).^37^ Histochemical detection of βgal activity in the brain of GM1 mice treated by thalamic injection of AAV-βgal shows strong staining throughout the parietal cortex and thalamus.^35^ Here, we observed a gradient of enhancement, which is most prominent in the ventral aspect of the brain (Figures 3G−3I). The apparent disparity between enzyme distribution and enhancement leads us to hypothesize that the kinetics of CSF penetration into the brain may be longer than the imaging time shown here. We anticipate that agent distribution into other brain structures may have increased over time. In line with this, correlation of enzyme activity and maximum MR enhancement for other brain regions (Figure S4) indicates that MR enhancement is mostly influenced by kinetics of CSF penetration into the tissues. For example, although enzyme activity of cerebellum and brainstem of the AAV-treated mouse (Figures S4D and S4E, filled red square) is less than two wild-type mice (open green diamond and circle), the MR enhancement of AAV treated is greater than wild type. This indicates that within the ∼2 h after contrast administration, the area with most CSF exposure/penetration shows greatest enhancement. However, the pattern of distribution may change at later time points after the probe contained in the CSF is able to penetrate the brain. The catalytically active enzyme in CSF, as suggested by contrast enhancement, represents a pool of bioavailable βgal for uptake by untransduced cells through receptor-mediated endocytosis. Although the activity of βgal is optimal in the low pH environment of lysosomes, it likely has catalytic activity at neutral pH. Therefore, enhancement in CSF and/or tissue (in interstitial fluid or lysosomes) is a direct representation of a bioavailable therapeutically relevant enzyme regardless of the contrast agent compartmentalization. Further experiments are required to determine the contribution to enhancement in the lysosome versus extracellular fluid.

Treatment of lysosomal storage diseases by systemic AAV administration can, in part, be monitored by enzyme levels in blood, but this is not always reflected in targeted peripheral tissues.^38^^,^^39^ Additionally, evaluation of efficacy in the central nervous system (CNS) by blood enzyme quantification is not reliable due to the presence of the blood brain barrier. This is especially true when AAV is administered directly into brain parenchyma or by CSF where measurement of enzyme levels in the blood is not representative of enzyme levels in the CNS. Therefore, we developed a technology that can directly inform on individual tissue enzymatic restoration, at levels ranging from supraphysiologic to 50% of normal. In this study, the βgal contrast agent exhibited sufficient sensitivity to detect levels anticipated to be therapeutic in GM1 patients. For lysosomal storage diseases, restoration of 10%–20% of normal enzyme level is sufficient to correct most aspects of the disease.^40^ Therefore, developing enzyme-activated contrast agents with sufficient sensitivity is of critical importance. i.p. administration of the contrast agent in a heterozygous mouse was sufficient to cause a slight but detectable increase (∼4%) in MR signal enhancement of the liver. For future experiments, we plan to administer the contrast agent i.v., and we anticipate this to increase the kinetics and magnitude of MR signal enhancement. In addition, modification of the chemistry to synthesize a more sensitive version of the contrast agent is ongoing.

Free Gd(III) ions are known to be toxic to biological systems, but it has been well established that suitable ligands that strongly bind the lanthanide form non-bioavailable and thus nontoxic safe complexes.^41^ Nephrogenic systemic fibrosis (NSF) is the most prominent toxicity associated with Gd(III) agents, and the medical community has evaluated Gd(III) agents in patients with renal failure and regularly publishes updated guidelines to minimize the risk.^42^^,^^43^ New guidelines have essentially eliminated this risk^44^, ^45^, ^46^ with updated Gd(III) contrast agents having an excellent safety profile, with severe adverse events in only 1 in 40,000 injections.^47^ As a result, approximately 40% of clinical MR scans today employ Gd (III) chelates for contrast imaging (>20 million/year). Future studies will include assessments of safety and tolerability of repeated administration to enable future translation to patients.

This is the first report of a contrast agent that informs on enzymatic activity in target tissues after AAV gene therapy. The high correlation between enzyme activity and enhancement encourages further exploration of this technology for eventual translation to patients. The chemical architecture of this platform allows development of highly specific MRI agents for detection of a large number of enzyme targets by replacing the substrate. As illustrated in Figure 1, this class of contrast agents uses an enzyme-specific substrate “arm” to block access of water to a Gd(III) ion, thus suppressing its MRI signal. In the presence of the enzyme, the arm of the contrast agent is cleaved, Gd(III) is exposed to water, and the MR signal is detectable. The enzyme-specific arm of this probe can be modified to mimic the structure of other enzymatic substrates, therefore altering specificity for that enzyme. For example, this technology has also been used to specifically react with β-glucuronidase and our group in the process of testing that agent in MPS mice.^48^ The data described here and the previously published report led to the statement of the generalizability of this technology to develop contrast agents specific for multiple diseases.^32^ Subsequent experiments exploring the pharmacokinetics/dynamics in GM1 mice, distribution in GM1 cat, and further correlation with CSF/brain enzymatic activity are in progress.

## Materials and methods

### Synthesis and characterization of the MR contrast agent

See Lilley et al.^32^ for the synthesis, *in vitro* and *in vivo* characterization, and MRI in mouse models of the contrast agent used in this study.

### Animals and treatment

All animal procedures were approved by the University of Massachusetts Medical School Institutional Animal Care and Use Committee (IACUC). Male and female, wild-type (n = 4), GM1 (n = 12), and heterozygous (n = 4) mice were used in these experiments. 6- to 8-week-old GM1 mice were treated by AAV gene therapy. The AAV9 vector encoded mouse lysosomal acid βgal under a CBA promoter, as described, carrying an expression cassette comprising a version of the CBA promoter. The vector was produced by triple transient transfection of HEK29T cells and purified by iodixanol gradient centrifugation as previously described.^49^ The vector was administered either intracranially (thalamus, unilateral, 10^10^ vg, n = 4) or i.v. (3 × 10^11^ vg, n = 4). Mice were anesthetized using ketamine (75 mg/kg) and dexmedetomidine (0.5 mg/kg), and AAV vector was injected unilaterally in the right thalamus using the following stereotaxic coordinates measured from bregma (in millimeters): AP: −2.0, ML: 1.5, and DV: −3.5, as previously described,^35^ at an infusion rate of 0.2 μL/min. Animals were recovered using atipamezole (1 mg/kg). i.v. administration of AAV9-βgal was performed via the tail vein.

### MRI and analysis

i.v. and thalamic-AAV-treated (n = 4 per group), age-matched wild-type or heterozygous and GM1 mice (n = 2 per group) were included for both routes of contrast administration. Age of mice at the time of imaging ranged between 4 and 6 months. Fresh βgal-responsive contrast agent (lyophilized powder) was diluted in saline right before MRI of each mouse. Mice were injected with βgal-responsive contrast agent at 0.06 mmol/kg in the lumbar intrathecal space (30 μL). Mice treated i.v. with AAV9-βgal received 0.16 mmol/kg of contrast agent i.p. (100 μL). MRIs were acquired in a Philips 3T scanner (Philips Ingenia; Philips Healthcare, Best, the Netherlands) using two custom-made solenoid T/R coils for imaging brain (22 mm internal diameter) and abdomen (27 mm internal diameter). For T_1_-weighted brain imaging a 2D turbo spin echo (TSE) pulse sequence with a TSE factor of 3 was used with the following parameters: repetition time = 600 ms, echo time = 10.2 ms, flip angle = 90°, number of signal averages = 2, field of view (FOV) = 30 mm × 30 mm, data acquisition voxel size = 0.2 mm × 0.2 mm, 16 slices with slice thickness = 1 mm, and slice gap = 0. Mice treated by thalamic injection of AAV9-βgal vector were imaged pre- and post-contrast (starting approximately 20 min after contrast administration) for at least 90 min using a dynamic scan with a dynamic interval of 123.6 (s).

For T_1_-weighted abdomen imaging (liver and kidney), a 3D respiratory-triggered, magnetization-prepared pulse sequence was used. This sequence consists of a leading saturated recovery pulse segment followed by a gradient echo imaging segment. The saturation recovery time was 600 ms, and the following parameters were used for imaging: repetition time = 10.0 ms, echo time = 5.3 ms, flip angle = 8°, number of signal averages = 3, FOV = 60 mm × 32 mm × 20 mm, and data acquisition voxel size = 0.2 mm × 0.2 mm × 1 mm. The abdomen of GM1 mice treated i.v. with AAV9- βgal was imaged pre- and post-contrast (immediately after i.p. contrast administration) for at least 90 min using dynamic scans with a dynamic gap of ∼10 min, which slightly varies for each dynamic scan based on the triggering signal received by the scanner from the respiratory gating system. The respiratory gating device used in this study was a MR-compatible control/gating module for small animal monitoring and a gating system (Model 1025; SA Instruments, Stony Brook, NY, USA). MR signal intensity was analyzed using ImageJ software (NIH). To quantify signal intensity over time in slices that contain frontal cortex, striatum, thalamus, and midbrain, the intensity of the selected region of interest (ROI) located at the ventral region of brain was acquired and normalized to the intensity of the masseter muscle (in the thalamus slice), which its signal did not significantly change over time (Figure S2). To quantify signal intensity over time in the abdomen (liver and kidney), signal intensity of selected ROIs was acquired and got normalized to the signal intensity of that ROI acquired at the first dynamic scan. To analyze MR signal intensity in livers, areas with partial volume effects from bile ducts or peritoneal fluid enhancement were excluded from the analysis. To sample a reasonable area of the liver tissue, the signal intensity was calculated in 4 ROIs, 2 ROIs per slice covering both the right and left lobes. The kinetics of multiple ROIs in the same mouse were similar; therefore, the ROIs located at the right lateral lobe of the liver were used for plotting kinetics of the signal in the liver and correlation analysis (Figure S5).

### Tissue preparation and enzyme assays

Mice were euthanized with an overdose of ketamine (375 mg/kg) and xylazine (37.5 mg/kg). Brain, spinal cord, liver, kidney, spleen, and abdominal fat were collected, frozen on dry ice, and stored at −80°C. The brain was divided in the cerebrum and cerebellum/brainstem. Tissues were homogenized in lysis buffer (0.2 M sodium acetate, 0.1 M sodium chloride, 0.1% Triton X-100, pH 4.3) using a TissueLyser II (QIAGEN, Germantown, MD, USA) with 5 mm stainless-steel beads at 20 Hz for 30 s for three pulses.

Lysates underwent three freeze thaws alternating between a dry ice-ethanol bath and 37°C water bath. Lysates were centrifuged at 20,000 × *g* for 15 min at 4°C, and the supernatant was transferred to a new microcentrifuge tube and stored at −80°C. Total protein content was measured by the QuickStart Bradford Protein Assay (Bio-Rad, Hercules, CA, USA) with serial dilutions of bovine serum albumin as the protein standard. Total βgal activity was measured in the brain (coronal block containing thalamus), liver, and kidney using 4-methylumbelliferyl-β-D-galactopyranoside (Sigma-Aldrich, St. Louis, MO, USA) as the synthetic fluorogenic substrate, specific for βgal. Total βgal activity was determined by measuring the release of 4-methylumbelliferone at excitation 360 nm and emission 460 nm and normalized to total protein concentration.

## Supplemental information

Document S1. Figures S1–S5

Document S2. Article plus supplemental information
