# Supplementary material for: Real-time MR tracking of AAV gene therapy with βgal-responsive MR probe in a murine model of GM1-gangliosidosis
Source: Mol Ther Methods Clin Dev. 2021 Aug 26;23:128–34. doi: 10.1016/j.omtm.2021.08.003 (PMC8517204; doi:10.1016/j.omtm.2021.08.003)
Supplement: Document S1. Figures S1–S5 [file mmc1.pdf]

OMTM, Volume *23*

Supplemental information

Real-time MR tracking of AAV gene therapy with βgal-responsive MR probe in a murine model of GM1-gangliosidosis

Toloo Taghian, Ana Rita Batista, Sarah Kamper, Michael Caldwell, Laura Lilley, Hao Li, Paola Rodriguez, Katerina Mesa, Shaokuan Zheng, Robert M. King, Matthew J. Gounis, Sophia Todeasa, Anne Maguire, Douglas R. Martin, Miguel Sena-Esteves, Thomas J. Meade, and Heather L. Gray-Edwards

Fig. S1.

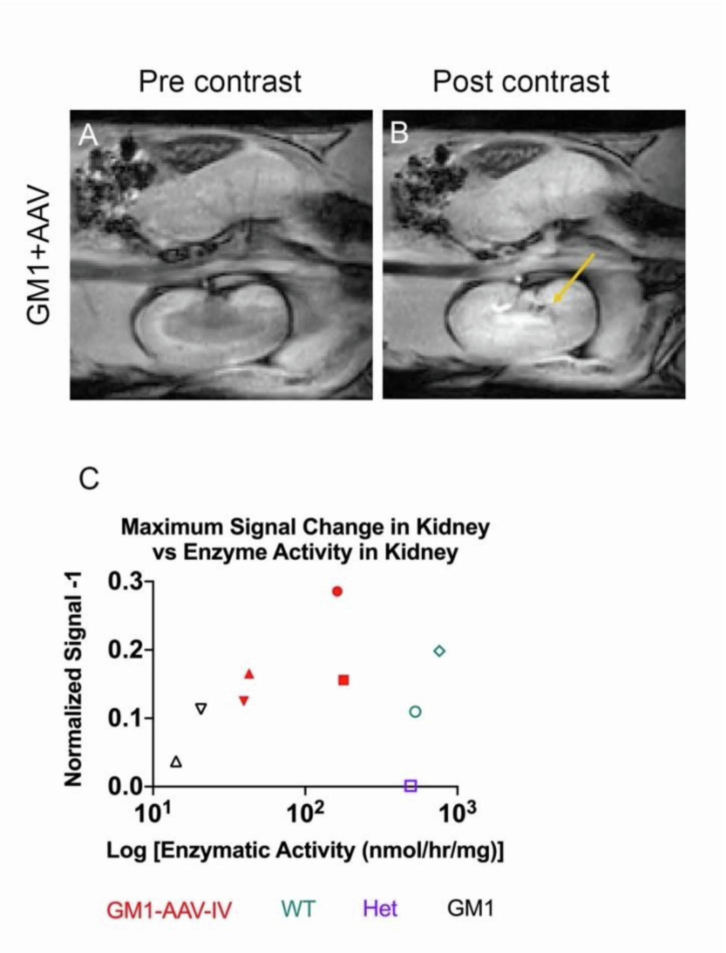

Fig. S1. Representative kidney images of an AAV treated GM1 mouse. (A) pre-contrast. (B) post-contrast administration. (C) Normalized MR signal in kidney does not correlate with kidney βgal enzyme activity (R^2=0.0001). Each symbol corresponds to individual mice. Correlation plot shows values for AAV treated GM1 mice (filled red squares, circles, and triangles; n=4), WT (wild type; open green diamonds and circles; n=2), Het (heterozygous; open purple square; n=1) and GM1 mice (open black triangles; n=2).

Fig S2.

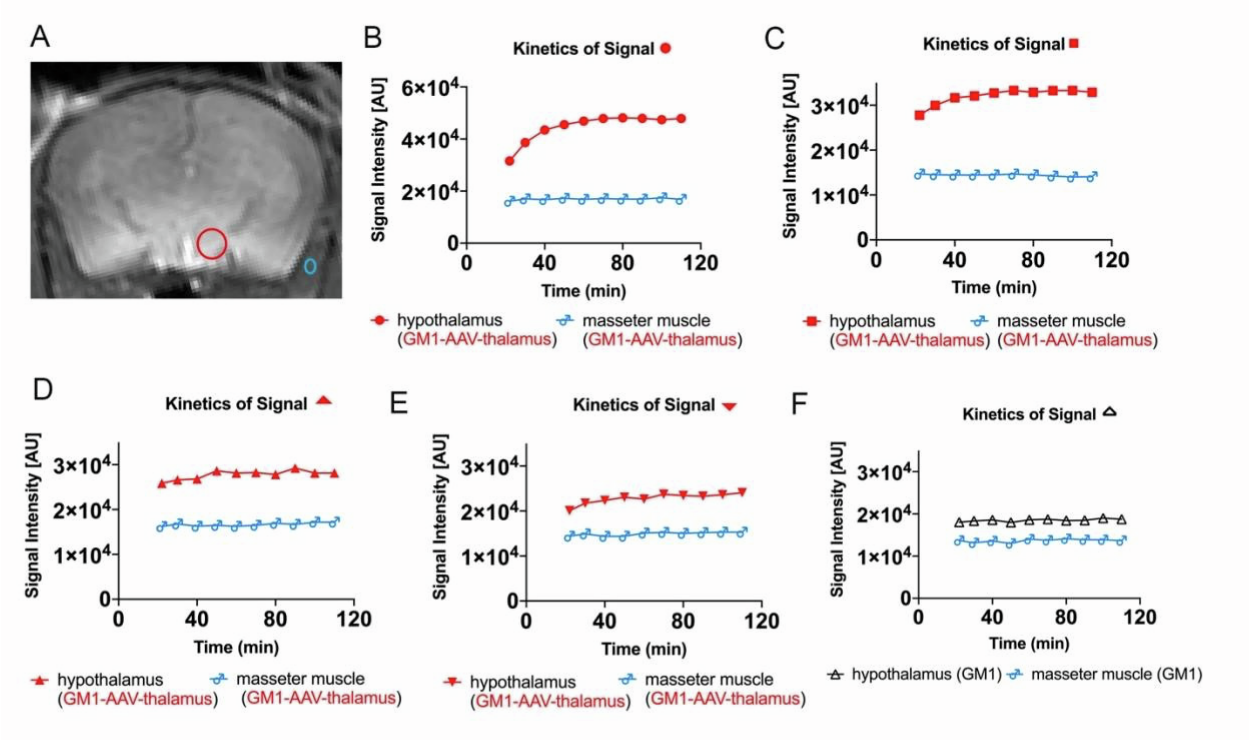

Fig. S2. Kinetics of MR signal in hypothalamus vs muscle. (A) Representative image of an AAV treated GM1 mouse thalamus. Red shows an ROI in hypothalamus and blue shows an ROI in masseter muscle used for normalization of signal acquired from thalami. (B-E) Signal intensity of four AAV-thalamus injected mice, quantified in hypothalamus and masseter muscle. Note that signal intensity of masseter muscle does not increase over time. (F) Signal intensity of a GM1 mouse quantified in hypothalamus and masseter muscle.

Fig S3.

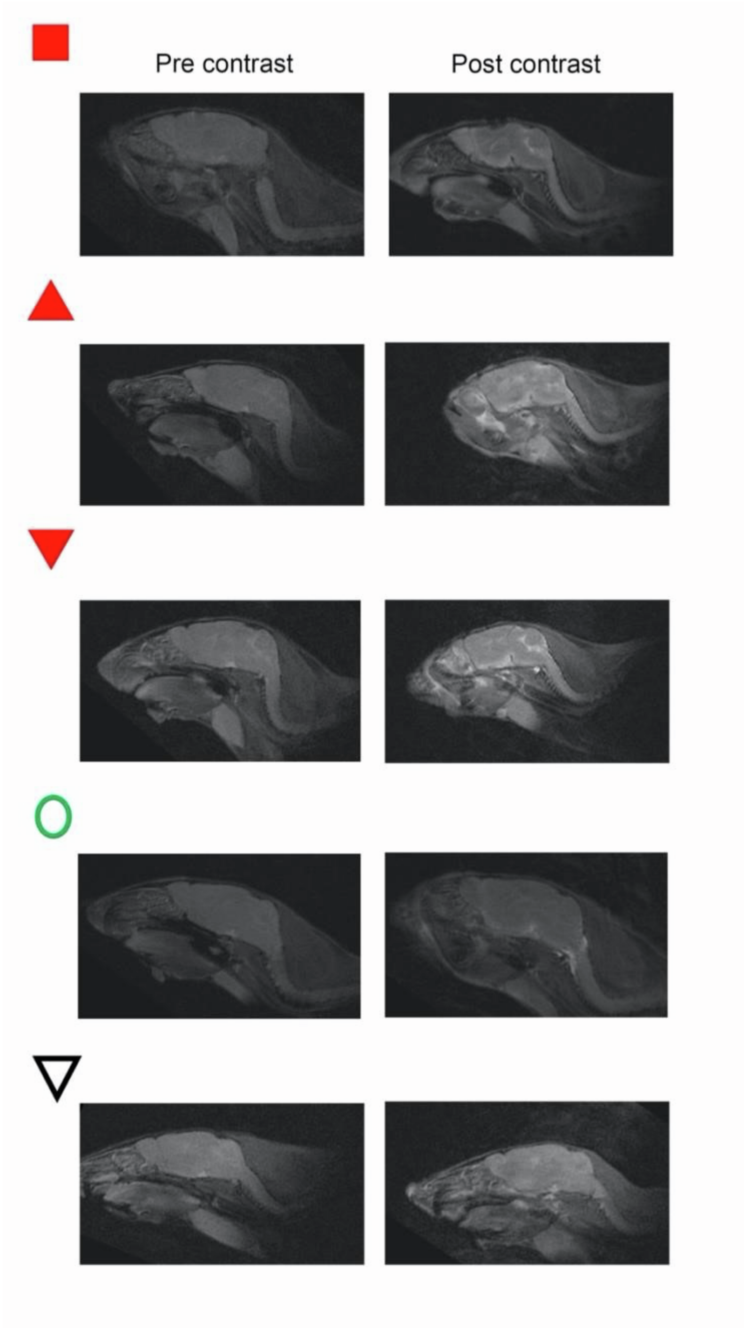

Fig. S3. **Proof of contrast injection into the intrathecal space.** Sagittal T_2w MRI pre- and post- contrast administration for three AAV-thalamus injected mice (filled red square, and triangles), one WT (wild type; open green circle) and one GM1 mouse (open black triangle). T_2 enhancement in the post contrast panel indicates successful injection in the intrathecal space. Geometrical shapes correspond to the mice shown in Fig.3.

Fig S4.

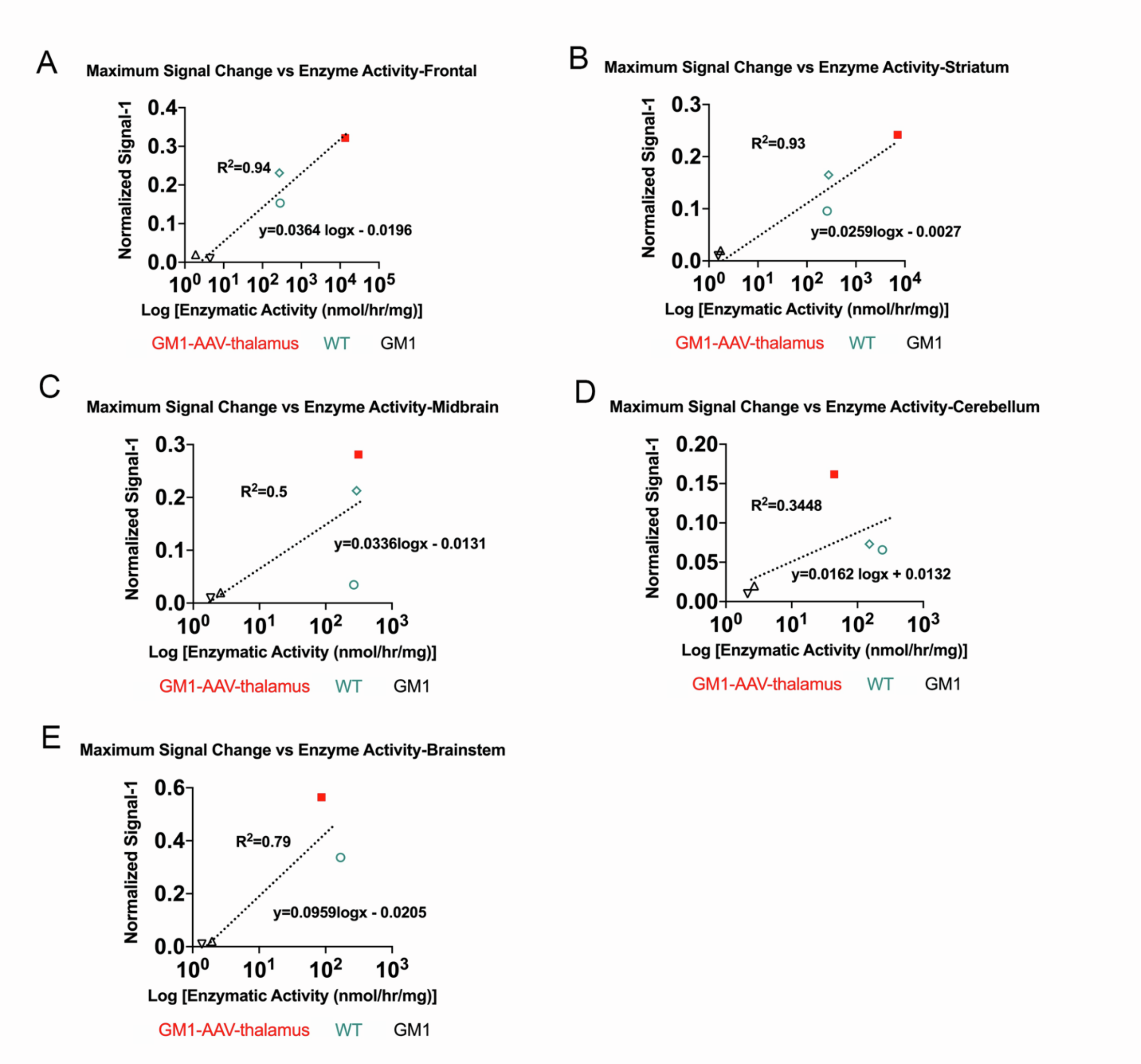

Fig. S4. **Correlation of Maximum MR enhancement with enzyme activity, quantified in each brain region.** MR signal for frontal, striatum, and midbrain was quantified in an ROI located at ventral region of brain (similar to the ROIs shown in Fig.3 J-L). ROIs for cerebellum and brain stem are similar to ROIs shown in Fig.3-A. Correlation performed for AAV treated GM1 mice (filled red squares), wild type (open green diamonds and circles; n=2) and GM1 mice (open black triangles; n=2). Three AAV treated GM1 mice are not represented because they didn't recover from anesthesia.

Fig S5.

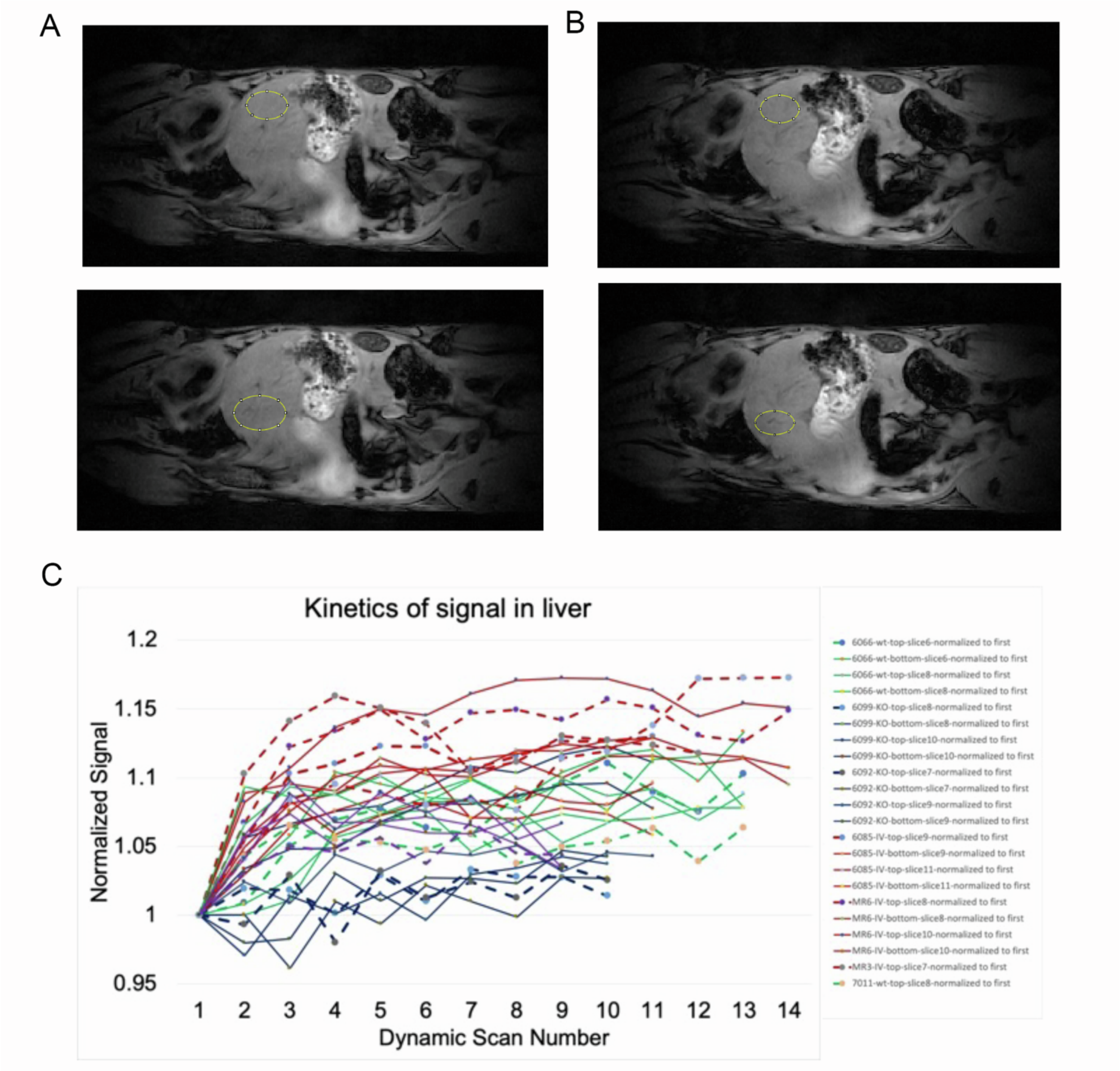

Fig. S5. **Criteria for ROI selection in liver.** To sample reasonable area of liver tissue, signal intensity was calculated in 4 ROIs, 2 ROIs per slice (A is slice 9 and B is slice 11 on the MRI) covering both the right and left lobes (shown on top and bottom images in A and B). The kinetics of multiple ROIs in the same mouse were quantified (C) which were similar between ROIs, therefore the ROI located at the right lateral lobe of liver were used for plotting kinetics of signal in liver and correlation analysis.

Movie S1.

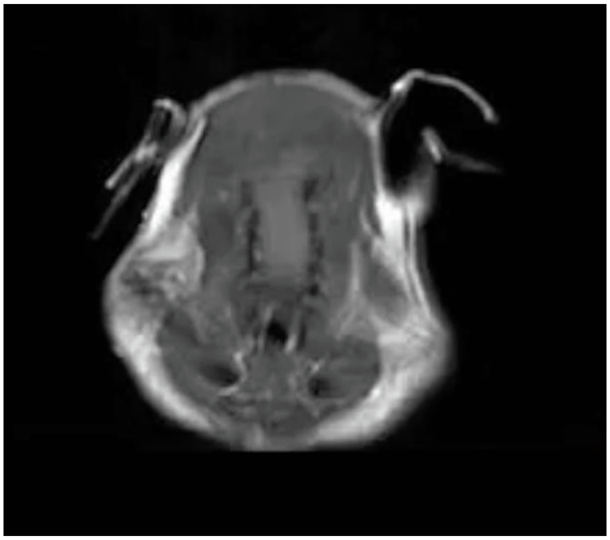

Movie S1. Activation of βgal-responsive contrast agent in the spinal canal and hindbrain. Movie represents an AAV-thalamus treated GM1 mouse. Post contrast MR imaging starts at 20 min after contrast administration and continues for 90 min (t=110 min).

Movie S2.

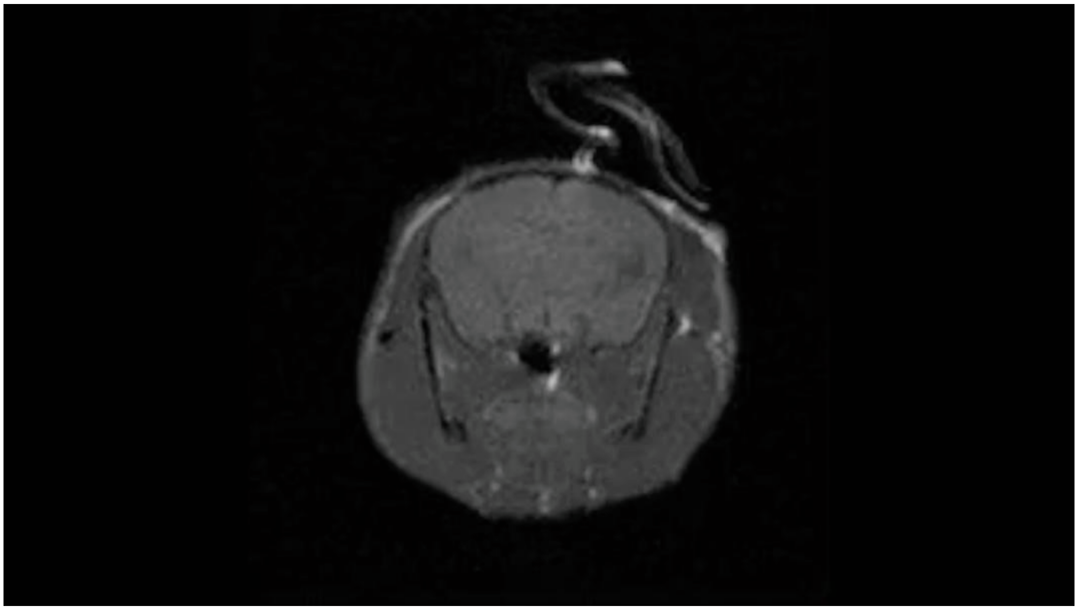

Movie S2. Activation of βgal-responsive contrast agent in the brain parenchyma. Movie represents an AAV-thalamus treated GM1 mouse. Post contrast MR imaging starts at 20 min after contrast administration. Note the gradual enhancement of the hypothalamus, thalamus, and cortex during the 90 min MR imaging.

Movie S3.

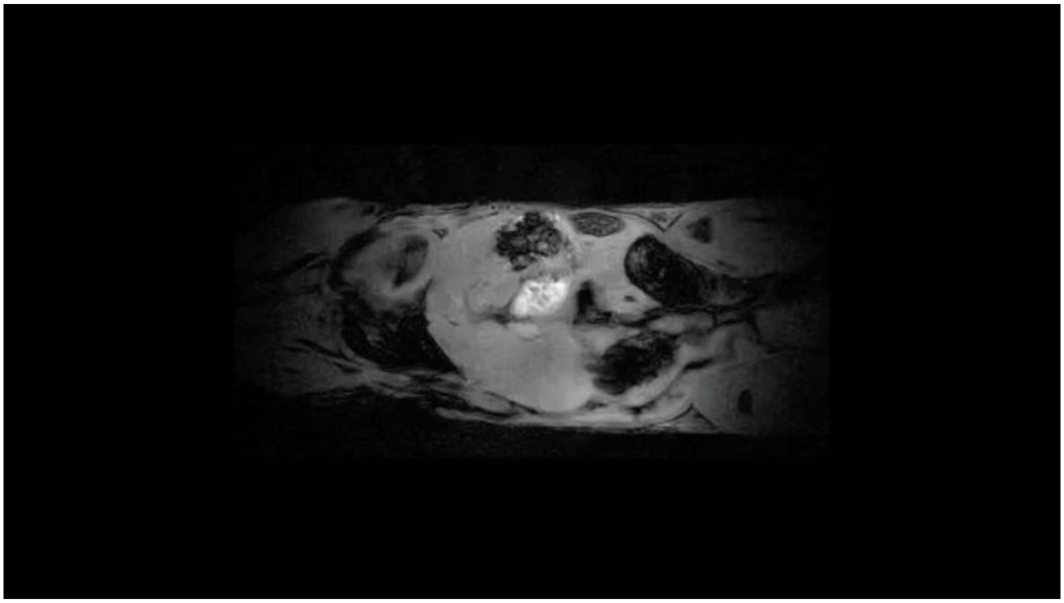

Movie S3. Activation of βgal-responsive contrast agent in the liver. Movie represents an intravenous AAV treated GM1 mice. Post contrast imaging starts quickly after contrast administration. Signal reaches a plateau 40-50 min after contrast administration.
